# Bone marrow-derived mesenchymal stem cells ameliorate severe acute pancreatitis by inhibiting oxidative stress in rats

**DOI:** 10.1007/s11010-022-04476-3

**Published:** 2022-05-27

**Authors:** Dongbo Zhao, Weidi Yu, Wangcheng Xie, Zhilong Ma, Zhengyu Hu, Zhenshun Song

**Affiliations:** 1 Department of General Surgery, Shanghai Tenth People’s Hospital, Clinical College of Nanjing Medical University, 301 Yanchang Middle Road, Shanghai, 200072 People’s Republic of China; 2 Department of General Surgery, Shanghai Tenth People’s Hospital, Tongji University School of Medicine, Shanghai, 200072 People’s Republic of China

**Keywords:** Severe acute pancreatitis, Mesenchymal stem cells, Nrf2, Oxidative stress

## Abstract

To investigate whether bone marrow mesenchymal stem cells (BMSCs) attenuate pancreatic injury via mediating oxidative stress in severe acute pancreatitis (SAP). The SAP model was established in rats. Phosphate buffered saline (PBS) or BMSCs were injected into the rats by tail veins. ML385 was used to down-regulate Nrf2 expression in rats. Pancreatic pathological score was used to evaluated pancreatic injury. Inflammatory-associated cytokines, serum lipase and amylase, levels of myeloperoxidase, malondialdehyde, reactive oxygen species and superoxide dismutase, as well as catalase activity were measured for injury severity evaluation. ML385 aggravates oxidative stress in SAP + ML385 group, compared with SAP + PBS group. BMSCs transplantation alleviated pancreatic injury and enhance antioxidant tolerance in SAP + BMSCs group, while ML385 administration weakened this efficacy in SAP + BMSCs + ML385 group. In addition, BMSCs promoted Nrf2 nuclear translocation via PI3K/AKT signaling pathway. Besides, BMSCs reduced inflammatory response by inhibiting NF-κB signaling pathway in SAP. BMSCs can inhibit oxidative stress and reduce pancreatic injury via inducing Nrf2 nuclear translocation in SAP.

## Introduction

Acute pancreatitis (AP) is a common digestive inflammatory disease. Approximately 20% of patients can still develop severe acute pancreatitis (SAP) with a mortality rate ranging from 7 to 30% [1–3]. The treatment of SAP remains relying on early fluid resuscitation and supportive care with limited success. Alternative treatment options are therefore needed.

Pathologically, SAP is the result of disruption of pancreatic homeostasis, characterized by acinar cell destruction and oxidative stress. Researches have shown that oxidative stress contribute to tissue damage during the early phases of SAP. On one hand, excess reactive oxygen species (ROS) produced by pancreatic acinar cells (PACs) can exacerbate vasoconstriction and vascular damage, leading to microcirculatory disturbance in pancreas, on the other hand, it activates the nuclear factor kappa B (NF-κB) signaling pathway in PACs, promoting the release of inflammatory cytokines and inducing the inflammatory cascade [4–6]. Previous studies have shown that inhibition of NF-κB can reduce inflammation in pancreatitis [7, 8]. Moreover, decreasing the production of ROS may be clinically valuable for the treatment of SAP.

Physiologically, excessive ROS are reduced by a series of antioxidants, most of which are regulated by nuclear factor erythroid-2-related factor 2(Nrf2). Nrf2 belongs to cap'n'collar subfamily of basic leucinezipper (cnc-bZip). In basal conditions, the Kelch-like ECH-associated protein 1 (Keap1) binds Nrf2 in the cytoplasm, giving rise to a ubiquitin ligase complex that ubiquitinates Nrf2 and subsequently degrades it. When under oxidative stress, increased production of ROS decreases the ubiquitin ligase activity, resulting in the dissociation of Nrf2 from Keap1 complex. The activated Nrf2 then translocate into the nucleus and binds to the promoter of antioxidant responsive element (ARE), upregulating the expression of antioxidants and anti-inflammatory molecules [9]. Fusco et al. found that hydroxytyrosol (HT), as an antioxidant phenol, was able to reduce pancreatic and intestinal injury in SAP mice, which was achieved in part via Nrf2 activation [10]. In addition, ML385 has been proved to exacerbate the course of acute pancreatitis in mice, which can block Nrf2 transcriptional activity by repressing binding of Nrf2 to the ARE promoter sequence [11, 12]. Considering the association between SAP and oxidative stress, Nrf2 may be a potential target for the treatment of SAP by enhancing resistance to oxidative stress.

Previous studies have confirmed that bone marrow-derived mesenchymal stem cells (BMSCs) could attenuate local pancreatic damage and systemic inflammation in SAP [13]. We also found that the levels of antioxidant enzymes in SAP rats treated with BMSCs were significantly increased, while the inflammatory infiltration in the pancreas was reduced [14, 15]. Thus, the homing BMSCs may modulate antioxidant release in SAP, while the underlying mechanism remains unclear. Hence, given the role of Nrf2 in antioxidant defenses, we speculated that the anti-oxidative effect of BMSCs in SAP was realized by regulating Nrf2 expression. This study was conducted to explore a new mechanism by which BMSCs attenuate SAP.

## Materials and methods

### Ethics statement

The animal experiments were approved by the animal ethics committee of Tongji University. All the animals were carried out in compliance with the guideline of the US National Institutes of Health for the care and use of laboratory animals (NIH publication No. 85-23, revised 1996). Surgeries were performed under sodium pentobarbital anesthesia; all the endeavors were made to minimize suffering.

### Isolation, culture and identification of BMSCs

BMSCs were isolated from wild-type male Sprague–Dawley rats (SD rats, 100–150 g, aged 4 weeks). In brief, bone marrow cells were isolated from the marrow cavity by flushing with DMEM-LG medium (Invitrogen Life Technologies, NY, USA). After centrifugation, the collected cells were cultured with medium-LG supplement with 10% fetal bovine serum (sigma-Aldrich, USA), 1% penicillin and streptomycin (C.C. Pro, Neustadt, Germany). Then, cells were incubated at 37 °C in humidified atmosphere with 5% CO_2_. Using flow cytometry for BMSCs identification. BMSCs from passages 3–5 were used for treatment. Our previous work has given a detailed description of the procedure above [16].

#### Establishment of SAP model and experimental groups

Wild-type Sprague–Dawley rats (SD rats, 200–250 g, aged 6 weeks) were purchased from Shanghai Laboratory Animal Co. Ltd (Shanghai, China). They were housed at 25 ℃ and 50% humidity with an alternating 12 h dark/light cycle, feeding with standard laboratory water and food. The SAP model were induced by retrograde pancreatic duct injection of 3% sodium taurocholate (NaT, 1 ml/kg body weight; 145-42-6, Sigma-Aldrich, St Louis, Missouri, USA) as previously described [17].

We randomly divided the rats into six groups (n = 6–8): normal control group (NC), Sham group (rats underwent the same operation with SAP rats without NaT injection), SAP + PBS group (SAP model treated with PBS), SAP + ML385 group (SAP model treated with ML385), SAP + BMSCs group (SAP model treated with BMSCs) and SAP + BMSCs + ML385 group (SAP model treated with BMSCs plus ML385). Either BMSCs(1 × 10^7^ cells/kg) or an equal volume of PBS were injected into the rats via tail vein within 6 h after SAP induction. ML385 (5 mg/kg body weight; HY-100523, MCE Co. Ltd., Shanghai, China) acted as Nrf2 inhibitor and was intraperitoneal injected 1 h before SAP induction [18]. Rats were euthanized 3 days after operations above.

#### Histopathology

The pancreatic tissues were sectioned at 5–6 μm (made 3 sections for each sample). Sections were stained with hematoxylin and eosin (H&E) and were evaluated according to the following four parameters: tissue edema, inflammatory cell infiltration, vacuolization, and cell necrosis. The observation and evaluation of the tissues were carried out separately by two observers. Table 1 showed the pathological scoring criteria and scoring method.

**Table 1:** Histological scoring for SAP

Score	Edema	Inflammatory cellular infiltration	Vacuolization	Necrosis
0	Absent	Absent	Absent	Absent
1	Diffuse expansion of interlobar septa	Around ductal margin	Periductal, < 5%	1–5 necrotic cells/HPF
2	Diffuse expansion of interlobular septa	In parenchyma, < 50% of lobules	Focal, 5–20%	6–10 necrotic cells/HPF
3	Diffuse expansion of interacinar septa	In parenchyma, 50–75% of lobules	Diffuse, 20–50%	11–15 necrotic cells/HPF
4	Diffuse expansion of intercellular septa	In parenchyma, > 75% of lobules	Severe, > 50%	> 15 necrotic cells/HPF

*HPF* High-power field. Pathological score = edema (0–4) + necrosis (0–4) + inflammatory cell infiltration (0–4) + vacuolization (0–4)

### Quantitative real-time PCR (qRT-PCR) assay

Total RNA was extracted from frozen pancreatic issues using Trizol reagent (Invitrogen). cDNA was synthesized with 1 μg of total RNA using PrimeScript RT Reagent Kit (TaKaRa). Using a KAPA SYBR FAST qPCR Kit (Kapa Biosystems) to perform qRT-PCR assay. GAPDH was used as the endogenous control. The primer sequences are as following: Nrf2, Forward (F): TCCCAGCAGGACATGGATTTG, Reverse (R): GCTGGCTGAATTGGGAGGAAT; and GAPDH, F: CGCTAACATCAAATGGGGTG, R: TTGCTGACAATCTTGAGGGAG.

### Biochemical analysis

The serum levels of amylase (mU/ml) and lipase (mU/ml) were assayed separately by colorimetric assay kit (BioVision, Milpitas, USA) according to the manufacture’s protocol. Cytokines in the serum including tumor necrosis factor (TNF)-α, interleukin(IL)-1β, IL-6, and IL-10 were measured by ELISA kits (Minneapolis, MN, USA, R&D Systems). Using agent kits (Jiancheng, Nanjing, China) to evaluate tissue levels of ROS, superoxide dismutase (SOD), malondialdehyde (MDA) and glutathione (GSH) and catalase (CAT) and myeloperoxidase (MPO) activity.

### Western blotting

Total proteins were extracted from the rat pancreas, while pancreatic nucleoproteins were extracted by using a Nuclear Protein Extraction Kit (Jiancheng, Nanjing, China). Protein concentrations were quantified using a BCA protein assay kit (Pierce BCA). Equal amount of proteins were separated on 8% SDS-PAGE gels and transferred onto the nitrocellulose membranes. The membranes were then incubated overnight at 4 °C with the following primary antibodies: Nrf2(dilution 1:1000, proteintech), Keap1(dilution 1:1000, CST), inhibitor of nuclear factor kappa B kinase β(IKKβ, dilution 1:1000, CST), NF-κB p65(dilution 1:1000, CST), phosphorylated phosphatidylinositol-4,5-bisphosphate 3-kinase(p-PI3K, dilution 1:1000, CST), phosphorylated protein kinase B(p-AKT, dilution 1:1000, CST), GAPDH (dilution 1:5000, proteintech), and laminB1(dilution 1:2000, Abcam). After incubated with the corresponding secondary antibodies for 2 h, the protein bands were visualized by using the Odyssey scanner (LI-COR Biosciences).

### Immunohistochemical analysis

3% hydrogen peroxide was used to incubate the dewaxed pancreatic tissue for 30 min. Then the sections were infiltrated in citrate buffer and boil at high pressure for antigen retrieval. Using anti- NF-κB p65(dilution 1:100, CST) and anti-Nrf2 antibodies (dilution 1:100, proteintech) and to stain the sections at 4 °C overnight. Then, washing the sections with PBS and applying the peroxidase-labeled secondary antibody at room temperature for 1 h. Finally, the sections were stained with 3,3′-diaminobenzidine tetrahydrochloride (DAB) and hematoxylin for visualization. The tissues of each sample were observed in five different fields by optical microscope at ×200.

### Statistical analysis

Experiments above are repeated independently at least three times. The collected data are shown as mean ± standard deviations (SD). Statistical analysis was processed by GraphPad Prism 8.0 (GraphPad Prism Software, CA, USA) and performed with an unpaired Student t-test or one-way ANOVA. A value of P < 0.05 was considered statistically significant.

## Results

### BMSCs ameliorate pancreas injury by regulating Nrf2

**Fig. 1:**
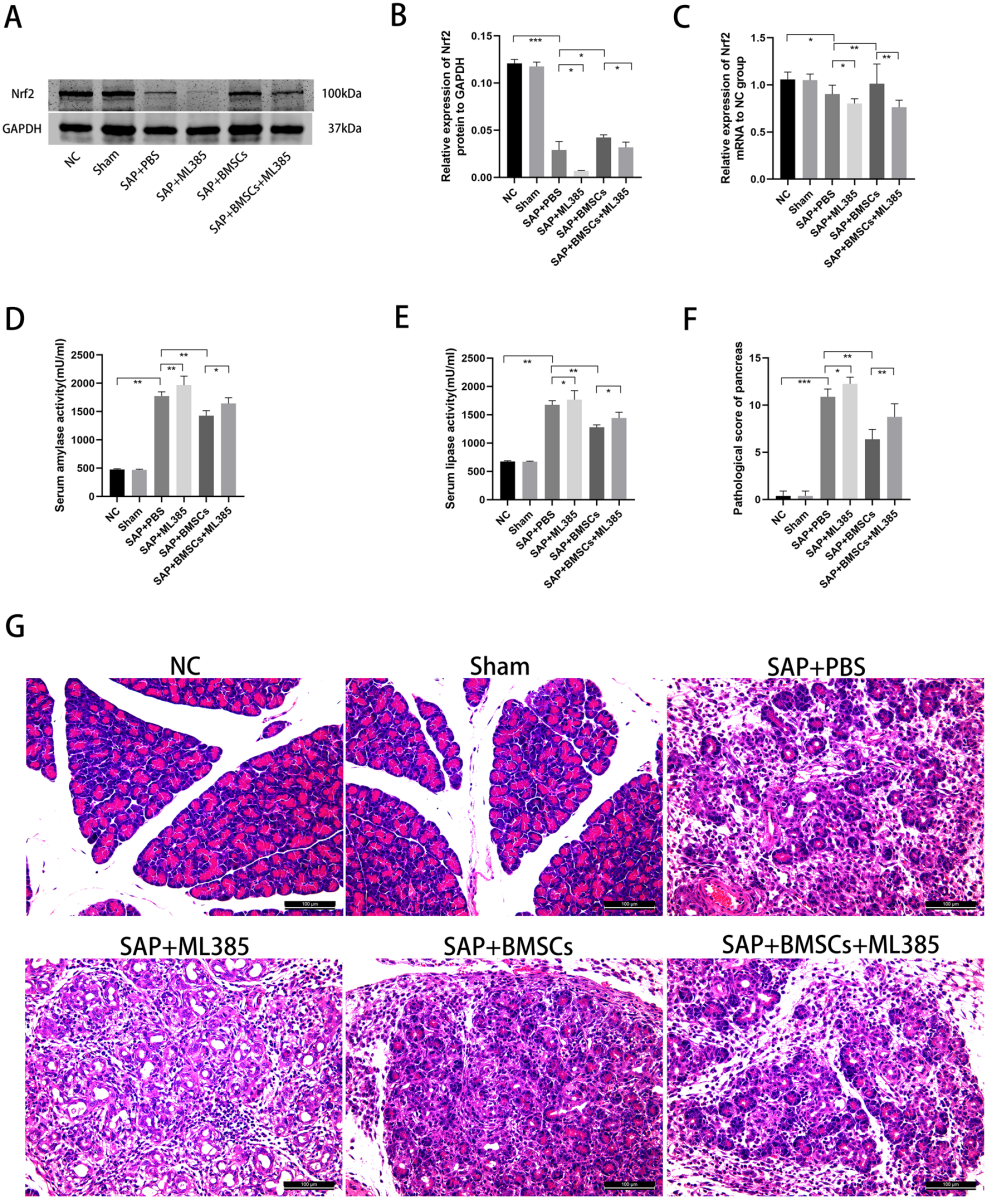
BMSCs could ameliorate SAP by regulating Nrf2. **A**–**C** ML385 inhibited the expression of Nrf2 in both SAP + ML385 and SAP + BMSCs + ML385 group. On the contrary, BMSCs up-regulated Nrf2 expression in injured pancreas, compared with SAP + PBS group. **D**–**G** BMSCs could significantly reduce the serum level of amylase, lipase and ameliorate tissue damage in SAP, compared with SAP + PBS group. However, the efficacy of BMSCs could be attenuated by ML385 as shown in SAP + BMSCs + ML385 group. Data are shown as mean ± SD for at least 3 separate experiments. (In **G**, × 200 magnification; n = 6 per group; *p < 0.05, **p < 0.01, and ***p < 0.001)

ML385 was injected as an inhibitor to down-regulate the expression of Nrf2. The expression of Nrf2 was significantly declined in SAP + ML385 group and SAP + BMSCs + ML385 group, indicating the reliability of ML385 (Fig. 1A, B). Compared with NC and Sham group, the serum levels of amylase and lipase were significantly higher than those in SAP groups (SAP + PBS group and SAP + ML385 group), as well as the pathological scores for injury severity evaluation. Moreover, the injection of ML385 exacerbated pancreatic injury in SAP + ML385 group, compared with SAP + PBS group. In addition, the injection of BMSCs obviously ameliorate pancreatic edema, infiltration, and acinar necrosis, the same applies for serum amylase and lipase, compared with SAP groups (Fig. 1A, B, D–G). Besides, the transplantation of BMSCs up-regulated the expression of Nrf2 (Fig. 1A, C), while ML385 weakened the protective effect of BMSCs in SAP (SAP + BMSCs group vs SAP + BMSCs + ML385 group). Therefore, we confirmed that BMSCs could ameliorate pancreas injury, while inhibition of Nrf2 could partially block this efficacy.

### BMSCs induce Nrf2 nuclear translocation via activating the PI3K/AKT pathway

**Fig. 2:**
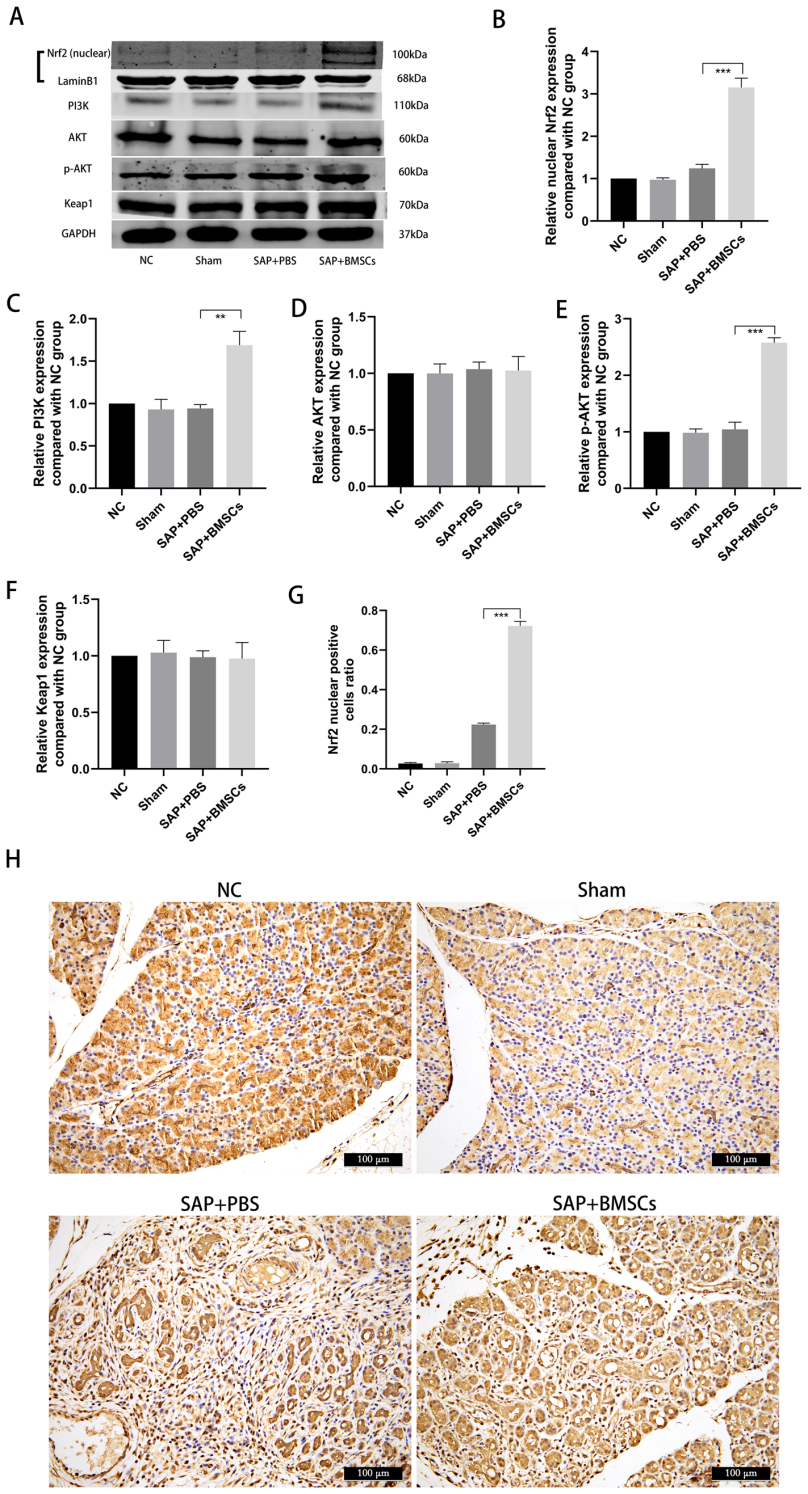
BMSCs induce Nrf2 nuclear translocation via the PI3K/AKT signaling pathway. **A**–**F** Western blot showed that BMSCs increased the total protein level of PI3K, and enhanced phosphorylation of AKT in SAP, whereas the total protein level of AKT was not significant changed. Besides, Nrf2 was notably increased in the nucleus in SAP + BMSCs group. **G**, **H** Immunohistochemistry was performed to confirm Nrf2 nuclear translocation induced by BMSCs. Data are shown as mean ± SD for at least 3 separate experiments. (In **H**, × 200 magnification; n = 6 per group; **p < 0.01 and ***p < 0.001)

To further explore the correlation of BMSCs and Nrf2, the nuclear level of Nrf2 was detected. BMSCs treatment increased Nrf2 translocation in the nucleus, whereas the expression of Keap1 was not significantly changed (Fig. 2A, F–H). In addition, upregulation of PI3K and p-AKT were also observed in SAP + BMSCs group, whereas total AKT expression remained unaltered (Fig. 2A–E). Previous studies have confirmed that PI3K/AKT signaling pathway is involved in Nrf2-mediated antioxidant activation and cell ferroptosis [19, 20]. Thus, we speculated that BMSCs could promote nuclear translocation of Nrf2 in SAP, which is achieved by activating the PI3K/AKT signaling pathway.

### BMSCs increase oxidative stress tolerance in SAP via inducing Nrf2 nuclear translocation

**Fig. 3:**
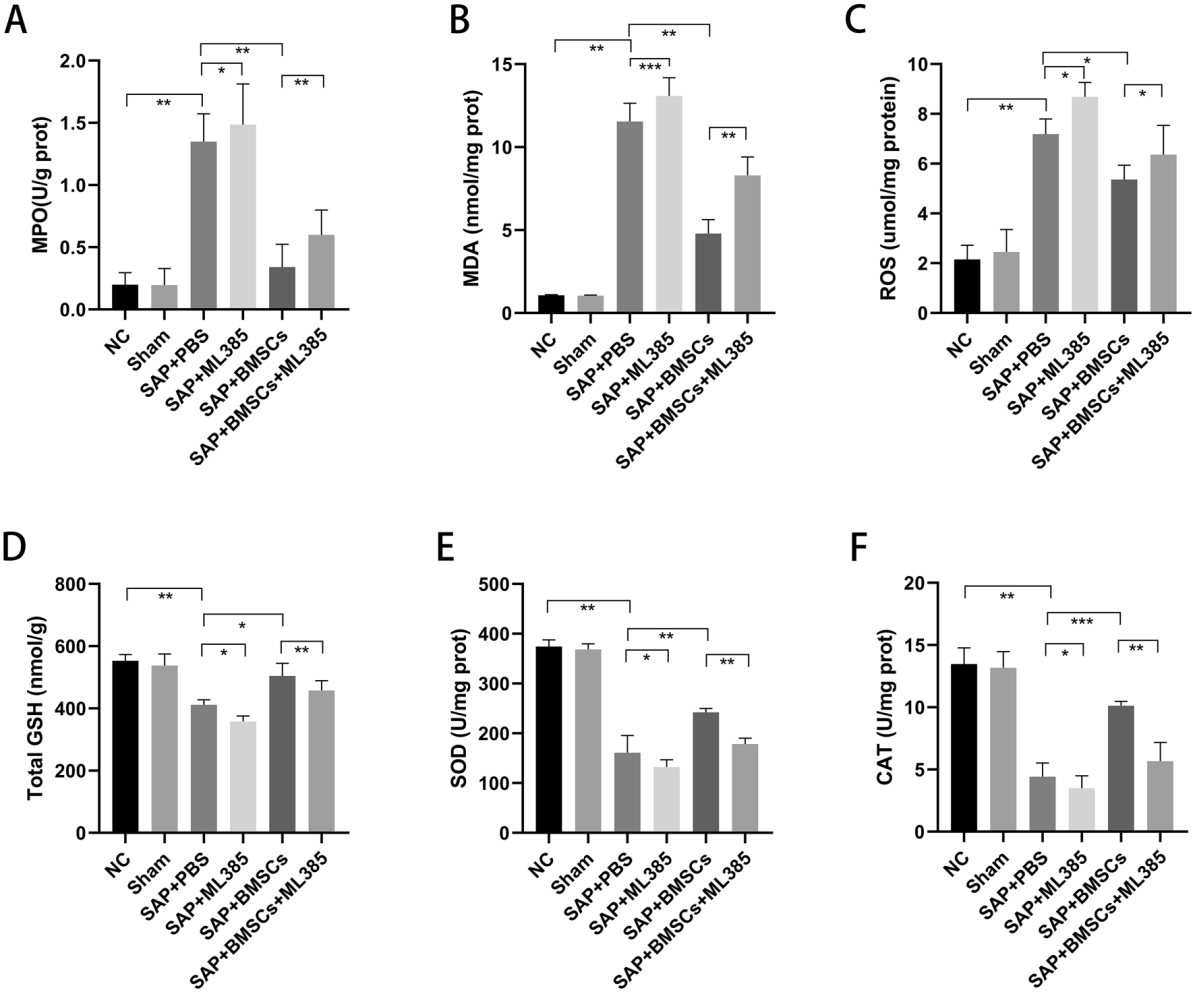
BMSCs increase oxidative stress tolerance in SAP. **A**–**F** The levels of MPO, MDA and ROS were increased and total GSH, SOD and CAT activity were reduced after SAP induction. BMSCs decreased the levels of MPO, MDA and ROS and enhanced GSH, SOD and CAT activity in SAP. However, intraperitoneal injection of ML385 could lessen these antioxidant effects as shown in SAP + BMSCs + ML385 group. Data are shown as mean ± SD for at least 3 separate experiments. (n = 6 per group, *p < 0.05, **p < 0.01, and ***p < 0.001)

A range of indexes were detected to assess antioxidant activity in SAP rats. The levels of MPO, MDA and ROS were increased and the level of GSH, SOD and CAT activity were reduced in SAP groups compared with the NC and Sham groups, but these were distinctly improved after BMSCs injection (Fig. 3A–F). Further, the levels of MPO, MDA and ROS were much higher in the SAP + BMSCs + ML385 group than those in the SAP + BMSCs group, whereas the level of GSH, SOD and CAT activity were decreased (Fig. 3A–F). Therefore, the protective effect of BMSCs on increasing oxidative stress tolerance can be blocked by ML385. In summary, BMSCs ameliorate SAP by enhancing pancreatic antioxidant activity via inducing Nrf2 nuclear translocation.

### BMSCs inhibit the NF-κB signaling pathway and reduce inflammation in SAP via inducing Nrf2 nuclear translocation

**Fig. 4:**
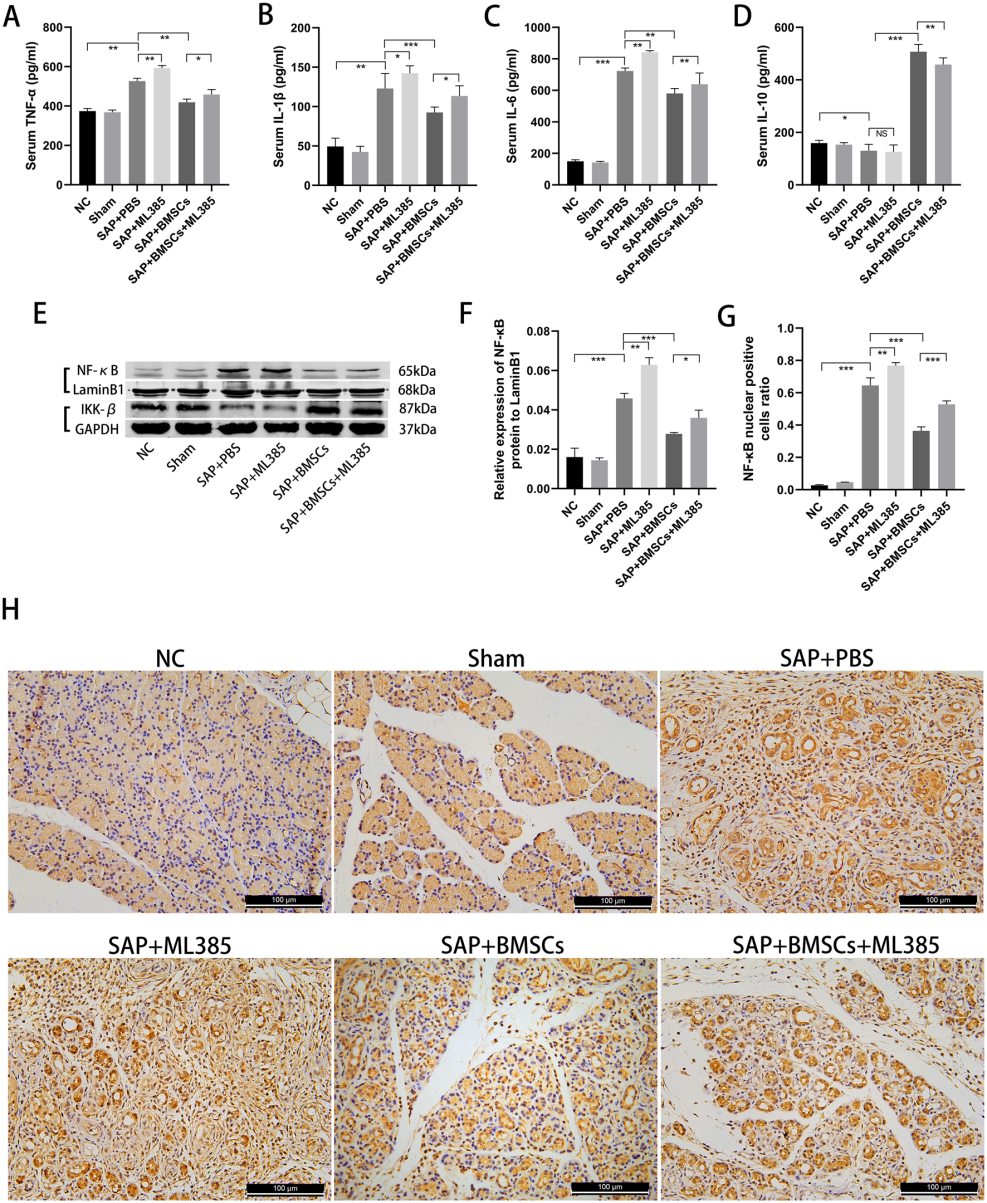
BMSCs inhibit the NF-κB signaling pathway and reduce inflammation in SAP via inducing Nrf2 nuclear translocation. **A**–**D** The expressions of pro-inflammatory cytokines (TNF-α, IL-1β and IL-6) were significantly decreased by BMSCs, while the expressions of anti-inflammatory cytokines (IL-10) were significantly increased. In addition, ML385 partially inhibited the anti-inflammatory effects of BMSCs. **E**, **F** Nuclear protein level of NF-κB p65 and total protein level of IKKβ were measured by Western blot. NF-κB p65 levels in the nucleus were decreased and the expression of IKKβ was increased after BMSCs treatment, while injection of ML385 increased NF-κB p65 levels in SAP + BMSCs + ML385 group. **G**, **H** Immunohistochemistry showed the nuclear enrichment of NF-κB p65. Data are shown as mean ± SD for at least 3 separate experiments. (In **H**, × 200 magnification; n = 6 per group; *p < 0.05, **p < 0.01, and ***p < 0.001)

Inflammatory-associated cytokines were measured to assess inflammation in SAP. The levels of pro-inflammatory cytokines (TNF-α, IL-6, and IL-1β) were significantly increased, whereas the anti-inflammatory cytokine (IL-10) level was decreased in SAP groups compared with NC and Sham group (Fig. 4A–D). Besides, BMSCs treatment could effectively attenuate pancreatic inflammatory severity. However, the inhibition of Nrf2 aggravated the inflammatory response in SAP + ML385 group, and the therapeutic effect of BMSCs was also mitigated in SAP + ML385 + BMSCs group (Fig. 4A–D).

Furthermore, we analyzed the expressions of NF-κB-associated genes (NF-κB p65 and IKKβ) in the pancreas. The expression of NF-κB p65 was observed increased after SAP induction, especially in SAP + ML385 group (Fig. 4E, F, H). As a suppressor protein of p65, IKKβ was also found decreased in SAP groups (Fig. 4E, G). After BMSCs transplantation, the expression of NF-κB p65 was down-regulated, as well as the expression of IKKβ was up-regulated in SAP + BMSCs group. However, inhibition of Nrf2 weakened the functional role of BMSCs in SAP + BMSCs + ML385 group (Fig. 4E–H). Therefore, we confirmed that BMSCs inhibited the NF-κB signaling pathway and reduced inflammation by inducing Nrf2 nuclear translocation in SAP.

## Discussion

SAP is an inflammatory disease marked by premature activation of digestive enzymes, inflammatory cell infiltration and tissue necrosis. Oxidative stress is one of the major pathways that contribute to PACs inflammation. Animal-based studies have shown that antioxidant treatment could ameliorate oxidative stress and decrease pro-inflammatory factor expression in SAP [21–23]. However, this treatment for SAP has been attempted with limited success in clinical [24]. A meta-analysis involving more than 3000 patients showed that antioxidant supplementation did not appear to prevent post-ERCP pancreatitis [25]. That is, patients of pancreatitis often derive less benefit from single antioxidant therapy. In addition, vascular endothelial injury is also a common pathologic finding in SAP, and thrombosis is a potentially fatal complication in patients undergoing SAP [26]. Inflammatory cell infiltration often contributes to increased vascular permeability and activation of coagulation cascades. In turn, the activated thrombin further stimulates inflammation creating a positive feedback loop [27, 28]. Given the complexity of the pathological mechanisms involved in SAP, a new comprehensive therapy is necessary.

BMSCs have the functions of self-renewal, multidirectional differentiation, immune regulation and paracrine [29]. Patil et al. found that MSC-derived exosomes could reduce inflammation, promote angiogenesis, and decrease infarct volume in myocardial ischemic injury, which confirmed the anti-inflammatory and reparative action of MSCs [30]. In addition, our previous studies have also shown that BMSCs reduce both local pancreatic injury and systemic inflammation by repairing vascular injury and inhibiting cell necroptosis [31, 32]. Therefore, BMSCs is expected to become the basis of a novel therapy for SAP. Oxidative stress plays a key role in the progression of SAP, while there is not much research on whether BMSCs therapy has antioxidant effects. In our study, the decreased expressions of antioxidants in SAP were reversed by BMSCs transplantation, indicating that BMSCs may protected sodium taurocholate induced SAP in rat by mediating antioxidants.

As a master regulator of inflammatory response, Nrf2 have been a research hotspot in recent years. The expression of Nrf2 is mainly carried out by the negative regulation of Keap1. Keap1 is a subunit of the E3 ubiquitin ligase based on Cullin3 (Cul3). During stress, the presence of electrophiles and ROS reduced the activity of E3 ligase in Keap1-Cul3 complex, which makes Nrf2 unstable and finally transfer into the nucleus [33, 34]. Wakabayashi et al. constructed a Keap1-deficiency mouse model that allowed Nrf2 to accumulate in the nucleus, eventually inducing cell protection [35]. In addition, activation of PI3K/AKT signaling pathway can also induce expression of Nrf2. In our study, BMSCs activated PI3K/AKT signaling pathway, followed by nuclear Nrf2 upregulation in acinar cells. Therefore, we confirmed that BMSCs could promote nuclear translocation of Nrf2 in SAP by activating the PI3K/AKT signaling pathway.

Whether AP progresses to SAP depends to some extent on the balance between oxidative stress and natural defense. Although the inflammation and the activation of trypsin are relatively independent without interaction, oxidative stress can participate in the process of both. Excess ROS released by NADPH oxidase can not only change the mitochondrial permeability transition pore (MPTP) directly, leading to apoptosis and necrosis, but also activate the NF-κB signaling pathway to initiate inflammatory response. Previous studies have proved the inhibitory effect of BMSCs on NF-κB signaling pathway [36]. Moreover, we found that this inhibition is partly achieved by promoting Nrf2 nuclear translocation.

**Fig. 5:**
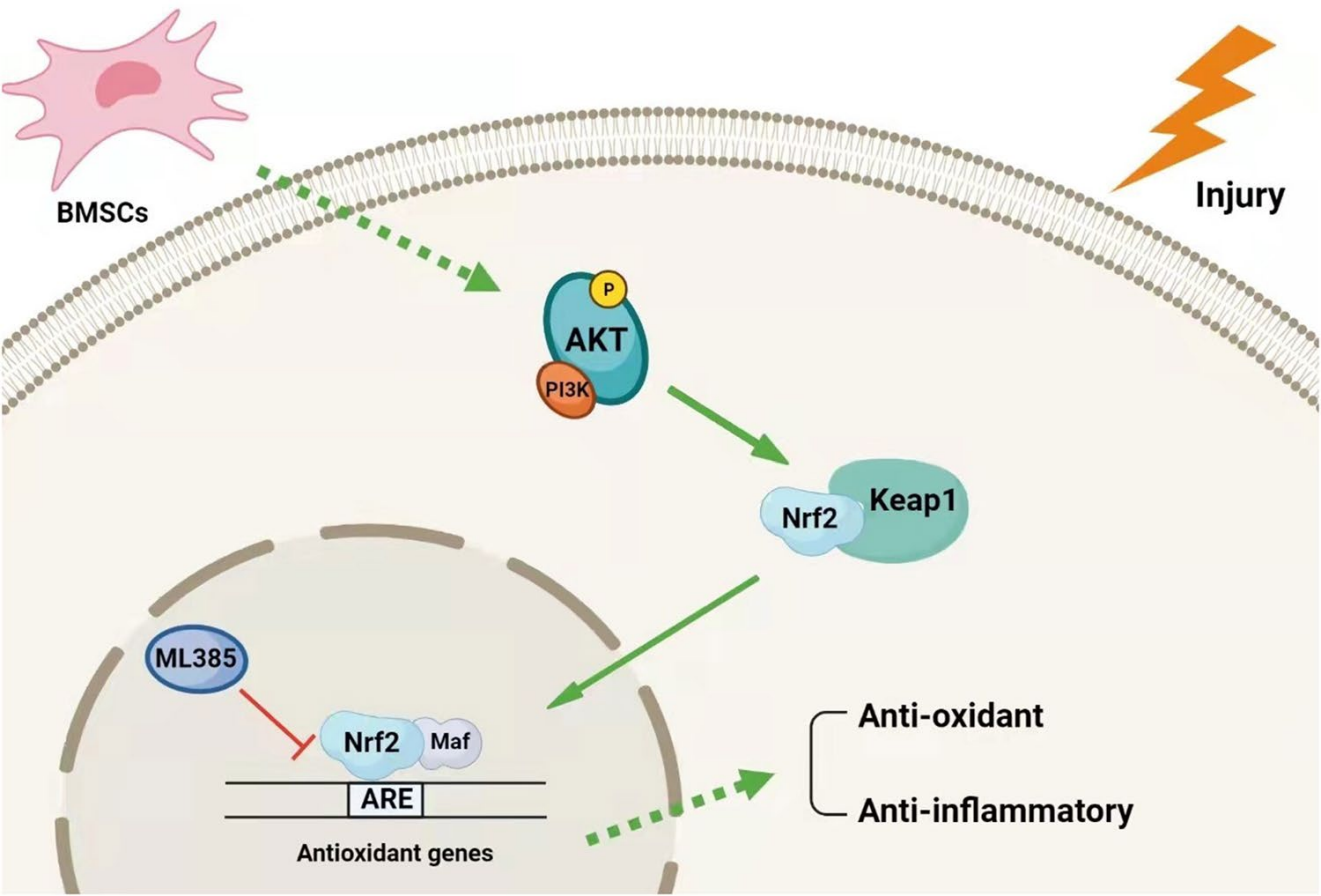
Possible mechanism of BMSCs therapy for SAP. Oxidative stress is boosted in pancreas as consequence of various injuries. The expression of Nrf2 will decreased in PACs, leading to antioxidant capacity decline in SAP. In addition, BMSCs may upregulate Nrf2 expression and induce Nrf2 nuclear translocation via the PI3K/AKT signaling pathway, which enhance antioxidant and anti-inflammatory effects in SAP

Although we have proved that BMSCs can mediate the oxidative stress reaction in the early stage of SAP. Whether the therapeutic effect can or will be sustained remains unknown and further monitoring of prognosis over a longer period is warranted. Nonetheless, our work has demonstrated that BMSCs can ameliorate SAP and inhibit oxidative stress by inducing Nrf2 nuclear translocation in rats (Fig. 5).

## Conclusions

The present study proved that BMSCs treatment effectively attenuated pancreatic injury severity in SAP rats. BMSCs reduced oxidative stress and enhanced antioxidant activity by inducing Nrf2 nuclear translocation via PI3K/AKT signaling pathway. In addition, BMSCs reduced inflammatory response in SAP by inhibiting the NF-κB signaling pathway. The results above suggest the potential therapeutic effect of BMSCs in SAP.

## Data Availability

All data generated or analyzed during this study are included in this published article [and its supplementary information files].

## Abbreviations

BMSCs: Bone marrow-derived mesenchymal stem cells
SAP: Severe acute pancreatitis
Keap1: Kelch-like ECH-associated protein 1
PBS: Phosphate buffered saline
ROS: Reactive oxygen species
MPO: Myeloperoxidase
SOD: Superoxide dismutase
MDA: Malondialdehyde
GSH: Glutathione
CAT: Catalase
ARE: Antioxidant responsive element
HPF: High-power field
MPTP: Mitochondrial permeability transition pore

## Code availability

Code availability not applicable to this article as no code were generated during the current study.
